# The Migration of Human Follicular Dendritic Cell-Like Cell Is Facilitated by Matrix Metalloproteinase 3 Expression That Is Mediated through TNF*α*-ERK1/2-AP1 Signaling

**DOI:** 10.1155/2021/8483938

**Published:** 2021-06-17

**Authors:** Hyo-Kyung Pak, Yong-Woo Kim, Bora Nam, A-Neum Lee, Jin Roh, Minchan Gil, Chaohong Liu, Yoo-Sam Chung, Chan-Sik Park

**Affiliations:** ^1^Department of Pathology, Asan Medical Center, University of Ulsan College of Medicine, Seoul, Republic of Korea; ^2^Institute for Life Sciences, Asan Medical Center, University of Ulsan College of Medicine, Seoul, Republic of Korea; ^3^Department of Pathology, Ajou University School of Medicine, Suwon, Republic of Korea; ^4^Department of Stem Cell and Regenerative Biotechnology, Konkuk University, Seoul, Republic of Korea; ^5^Department of Pathogen Biology, Tongji Medical College, Wuhan, China; ^6^Department of Otolaryngology, Asan Medical Center, University of Ulsan College of Medicine, Seoul, Republic of Korea

## Abstract

Follicular dendritic cells are important stromal components of the germinal center (GC) and have pivotal roles in maintaining the GC microenvironment for high-affinity antibody production. Tumor necrosis factor-*α* (TNF*α*) is essential for the development and functions of follicular dendritic cells. Despite the importance of follicular dendritic cells in humoral immunity, their molecular control mechanisms have yet to be fully elucidated due to the lack of an adequate investigation system. Here, we have used a unique human primary follicular dendritic cell-like cell (FDCLC) to demonstrate that the migration of these cells is enhanced by TNF*α*-mediated metalloproteinase 3 (MMP3) expression. MMP3 was found to be highly expressed in normal human GCs and markedly upregulated in human primary FDCLCs by TNF*α*. TNF*α* induced ERK1/2 phosphorylation and the transcription of *MMP3* through AP1. TNF*α* treatment increased FDCLC migration, and a knockdown of MMP3 significantly reduced the TNF*α*-induced migration of FDCLCs. Overall, we have newly identified a control mechanism for the expression of MMP3 in FDCLCs that modulates their migration and may indicate an important role in GC biology. Since GCs are observed in the lesions of autoimmune diseases and lymphomas, targeting the MMP3/TNF*α*-mediated migration of stromal cells in the B cell follicle may have great potential as a future therapeutic modality against aberrant GC-associated disorders.

## 1. Introduction

The germinal center (GC) is critical for the production of high-affinity protective antibodies and plays an important role in various immunologic disorders including autoimmune diseases and lymphomas [1, 2]. Follicular dendritic cells (FDCs) are the major stromal components of GCs and are essential for their formation and maintenance. These cells form network-like structures with their long dendrites and provide crucial survival and differentiation signals to GC-B cells [3, 4]. FDCs are developed and maintained by lymphotoxin *α*1/*β*2 and tumor necrosis factor-*α* (TNF*α*), which are produced from the B cells within the GC [5]. The spreading of the GCs together with FDCs is one of the important pathologic features of lymphoid diseases such as rheumatoid arthritis and follicular lymphomas [6]. Many of the molecular mechanisms underlying the actions of FDCs remain to be identified because of the lack of adequate investigation systems for these cells, particularly in relation to their migration.

TNF*α* is a pleiotropic cytokine that has pivotal roles in prolonged inflammation and autoimmune diseases [7, 8]. Blocking TNF*α* signaling has become the major treatment for those diseases [9]. Clinical treatments with the TNF*α* inhibitor etanercept have been reported to reduce the GC number in rheumatoid arthritis patients [10]. TNF*α* is also required for the development of the FDC network and the GC. Mice deficient in the TNF receptor fail to develop the GC and FDC clusters [11]. TNF*α* knockout mice have a B cell follicle deficiency, defects in FDC network organization, and impaired humoral immunity [12]. In addition, several studies have reported that TNF*α* signaling induces the expression of matrix metalloproteinases (MMPs). MMP1 is increased by TNF*α* in human fibroblasts [13]. TNF*α* also induces MMP3 expression in nucleus pulposus cells [14] and synovial fibroblasts [15]. MMP3 degrades ECM components during tissue remodeling and can thus be implicated in GC organization and FDC migration [16, 17].

Here, we investigate the molecular mechanisms underlying FDC migration using a primary human FDC-like cell (FDCLC) system. We show from our present analyses that MMP3 expression is required for TNF*α*-invoked FDCLC migration and is induced through TNF*α*-extracellular signal-regulated kinase 1/2- (ERK1/2-) activator protein-1 (AP1) signaling. This result sheds new light on the control of GC-related autoimmune diseases and FDC-associated fatal disorders including AIDS, prion infection, and follicular lymphoma. To our knowledge, this is the first report to describe the molecular processes that drive the migration of human primary FDCLCs.

## 2. Materials and Methods

### 2.1. Reagents and Antibodies

Hank's Balanced Salt Solution (HBSS, 14025092), Opti-Minimal Essential Medium (Opti-MEM; 31985070), and Roswell Park Memorial Institute (RPMI) 1640 medium (11875093) were purchased from Thermo Fisher Scientific (Waltham, MA). Bovine serum albumin (BSA; A9418) was obtained from Sigma-Aldrich (St. Louis, MO), and collagenase IV (LS004188) was sourced from Worthington Biochemical Corporation (Lakewood, NJ). U0126 (S1102), PD98059 (S1177), SP600125 (S1460), and SB203580 (S1076) were purchased from Selleckchem (Houston, TX). Recombinant human TNF*α* (300-01A) was obtained from PeproTech (Rocky Hill, NJ). Ficoll-Paque PLUS (17-5442-02) was purchased from GE Healthcare (Marlborough, MA). Mouse anti-human MMP3 antibody (Ab; 1B4, sc-21732) and mouse anti-human *β*-actin (C4, sc-47778) were obtained from Santa Cruz Biotechnology, Inc. (Dallas, TX). Rabbit antibodies for the following proteins and phosphoproteins were purchased from Cell Signaling Technology (Beverly, MA): phospho-p44/42 mitogen-activated protein kinase (MAPK; T202/Y204) (9101), p44/42 MAPK (Erk1/2) (9102), phospho-SAPK/c-Jun N-terminal kinase (JNK) (T183/Y185) (9251), SAPK/JNK (9252), phospho-p38 MAPK (T180/Y182) (9211), and p38 MAPK (9212).

### 2.2. Isolation of Human FDCLCs

To investigate the molecular mechanism of FDCLC migration, we recently established isolation and culture methods for these cells derived from normal pediatric tonsil tissues. Human tonsils were obtained from routine tonsillectomies. Primary FDCLCs were then extracted from these tonsils via mechanical disruption and enzymatic digestion as previously described [18]. Briefly, specimens were cut into small fragments, placed in HBSS containing 2 mg/mL of collagenase IV at 37°C for 15 min. The released cells were collected and subjected to Ficoll gradient centrifugation. The interface layer that contained the FDCLCs was then collected, and the cells were centrifuged at 200 rpm for 10 min at 4°C over a discontinuous gradient of 7.5% and 3% BSA. FDCLC-enriched fractions were then carefully collected from the interface. The cells were subsequently washed with HBSS and cultured on tissue culture dishes. Nonadherent cells were removed and adherent cells replenished with fresh medium every 3-4 days. Adherent cells were trypsinized when confluence was attained.

### 2.3. Ethical Approval for the Use of Human Tissues

The use of human tonsils that were obtained as leftover material after tonsillectomies was approved by the institutional review board of Asan Medical Center, Seoul, Korea (approval number, 2013–0864). Informed consent was waived because there was no additional risk to the participants and their identities were anonymized and completely delinked from unique identifiers.

### 2.4. Microarray Analysis

Total RNAs from TNF*α*-stimulated FDCLCs were purified using NucleoSpin RNA (740955, Macherey-Nagel, Düren, Germany). The purity and integrity of the isolated RNAs were evaluated by denaturing gel electrophoresis, via the OD 260/280 ratio, and by analysis on an Agilent 2100 Bioanalyzer (G2939BA, Agilent Technologies, Palo Alto, CA). cRNAs were generated from total RNA extracts using an Ambion Illumina RNA Amplification Kit (AMIL1791, Ambion, Austin, TX) and hybridized to Human HT12 Expression v.4 Bead Arrays in accordance with the manufacturer's instructions (Illumina, Inc., San Diego, CA). Array signals were detected using Amersham fluorolink streptavidin-Cy3 (GE Healthcare Bio-Sciences, Little Chalfont, UK) and an Illumina BeadArray Reader Confocal Scanner.

### 2.5. Secreted Protein Analysis by Luminex

FDCLCs were seeded in a 24-well plate at 2 × 10^4^ cells/mL/well to which 20 ng/mL of TNF*α* was added 24 h later. The culture supernatants were harvested after another 24 h, and the detached cells were removed by centrifugation. The supernatants were stored at -70°C. Three independent samples were prepared and pooled for Luminex analyzer (Rules-Based Medicine, Austin, TX).

### 2.6. Immunohistochemistry

Human tonsils were embedded with paraffin and sectioned at a 4-micron thickness. These tissue sections were then deparaffinized, rehydrated, and heated with ammonium citrate (pH 6.0) for 10 min. The sections were next incubated with a Dako protein block for 5 min. Primary antibodies against human MMP3 were incubated with the sections overnight at 4°C. MMP3 expression was visualized the next day using EnVision+ System-HRP Labelled Polymer Anti-Mouse (K4001, Agilent Technologies) for 1 h at RT, and counterstained with hematoxylin. The expression of MMP3 in human GCs was assessed under a Motic BA400 microscope.

### 2.7. Quantitative PCR (qPCR)

cDNA was synthesized from total RNA preparations using an iScript cDNA Synthesis Kit (1708891, Bio-Rad Laboratories, Hercules, CA). Quantitative PCR was then performed using the Power SYBR Green PCR Kit (4367659, Applied Biosystems, Foster City, CA), in accordance with the manufacturer's guidelines. Relative transcript levels were calculated using the comparative Ct method [19], and the expression of S18 was used as an internal control. The following gene-specific primers were used: Mmp3, 5′-ATTCCATGGAGCCAGGCT-3′ (sense) and 5′-CATTTGGGTCAAACTCCAACTGTG-3′ (antisense); S18, 5′-TTTGCGAGTACTCAACACCAACA-3′ (sense) and 5′-CCTCTTGGTGAGGTCAATGTCTG-3′ (antisense).

### 2.8. Western Blotting

For western blotting analysis, protein extracts from FDCLCs were separated on 12% SDS-polyacrylamide gels and electrophoretically transferred to an Immun-Blot PVDF Membrane (162-0177; Bio-Rad Laboratories). The membrane was then blocked for 1 h with 5% BSA and incubated overnight with MMP3 Abs. Unbound primary Abs were removed by washing 3 times with TBS/0.1% Tween 20, and the membrane was then incubated with horseradish peroxidase-conjugated anti-mouse secondary Abs (diluted 1 : 3,000 in TBS/0.1% Tween 20). Signals were visualized using an ImageQuant LAS 4000 biomolecular imager (GE Healthcare Life Sciences). *β*-Actin was used as an internal control.

### 2.9. Enzyme-Linked Immunosorbent Assay (ELISA) for MMP3

Secreted MMP3 was assayed using a Human Total MMP-3 Quantikine ELISA Kit (DMP300, R&D systems, Minneapolis, MN). Briefly, FDCLCs were seeded into 24-well plates at 2 × 10^4^ cells/mL/well and treated with 20 ng/mL of TNF*α*. The culture supernatants were harvested, and the MMP3 level was assayed according to the manufacturer's protocol.

### 2.10. Luciferase Reporter Assays for Human Mmp3

Reporter plasmids for the human Mmp3 promoter were constructed as follows: the Mmp3 proximal promoter region (–1370 to +202) was amplified by PCR from FDCLC genomic DNA and inserted into pGreenFire (TR010PA-1, System Biosciences, Palo Alto, CA). As FDCLCs exhibit very low transfection efficiency, and also low transduction by general lentiviral transduction methods, the previously described measles virus glycoprotein-displaying lentivirus transduction system was used [20]. All virus clones (mCMV, hMMP3p, AP1-3, and hMMP3p-mut) were produced at the same time, and the virus copy number was measured by p24 qPCR. FDCLCs were transduced using equivalent amounts of the viral clones and subjected to reporter assays using the Luciferase Assay System (Promega Corporation, E1501, Madison, WI). Briefly, the transduced FDCLCs were plated into 24-well plates (5 × 10^4^ cells/well), and the cells were stimulated with 20 ng/mL of TNF*α* and lysed. Luminescence was then measured with a VICTOR X3 Multilabel Plate Reader (PerkinElmer, 2030-0050, Waltham, MA).

### 2.11. Cell Migration Assay

To investigate the role of MMP3 in the migration of FDCLCs, we conducted a migration assay for these cells using a Matrigel-coated Transwell system. Transwell inserts (Transwell Permeable Supports with an 8.0 *μ*m polycarbonate membrane and 6.5 mm insert; 3422, Corning, Corning, NY) were first coated with Matrigel (354277, Corning) overnight at 37°C. Then, we resuspended1 × 10^5^FDCLCs in 100 *μ*L of RPMI 1640 containing 0.1% BSA, and we added these to the Matrigel-coated upper chamber of the Transwell inserts. Subsequently, 800 *μ*L of RPMI 1640 containing 10% FBS was added to the lower chamber. The cells were allowed to migrate through the Matrigel for 18 h, and the cells remaining above the Transwell membrane were removed by gentle scraping with a swab. The migrated cells on the bottom of the Transwell insert were fixed and stained using a Hemacolor Rapid blood smear kit (111661, Merck, Burlington, MA). The stained Transwell inserts were air-dried, and the migrated cells were counted under a microscope.

### 2.12. Gene Knockdowns via siRNA

To knockdown MMP3 expression, siRNA complementary to human Mmp3 was introduced into FDCLCs using Lipofectamine RNAiMAX (13778150, Thermo Fisher Scientific). Briefly, 5 pmol of the siRNAs was mixed with 1.5 *μ*L of Lipofectamine RNAiMAX in 50 *μ*L of Opti-MEM. The mixture was then added to 5 × 10^4^ cells/well/24-well plate. The cells were incubated for 3 days, and the reduction of MMP3 expression was confirmed by qPCR and ELISA. The double-stranded siRNAs were synthesized by Genolution Pharmaceuticals (Seoul, Korea) with the following sequences: Scr (negative control), 5′-CCUCGUGCCGUUCCAUCAGUAGUU-3′ (sense) and 5′-UACCUGAUGGAACGGCACGAGGUU-3′ (antisense); hMmp3i, 5′-GAGUUUGACCCAAAUGCAAAGAAAGUU-3′ (sense) and 5′-CUUUCUUUGCAUUUGGGUCAAACUCUU-3′ (antisense).

### 2.13. Statistical Analysis

All experiments were repeated at least three times. Statistical significance was analyzed using paired *t*-tests and GraphPad Prism software (version 6). Data were expressed as the mean value ± standard deviation. A *p* value of < 0.05 was considered statistically significant.

## 3. Results

### 3.1. Human FDCLCs in the GC Express MMP3 via TNF*α* Induction

**Figure 1:**
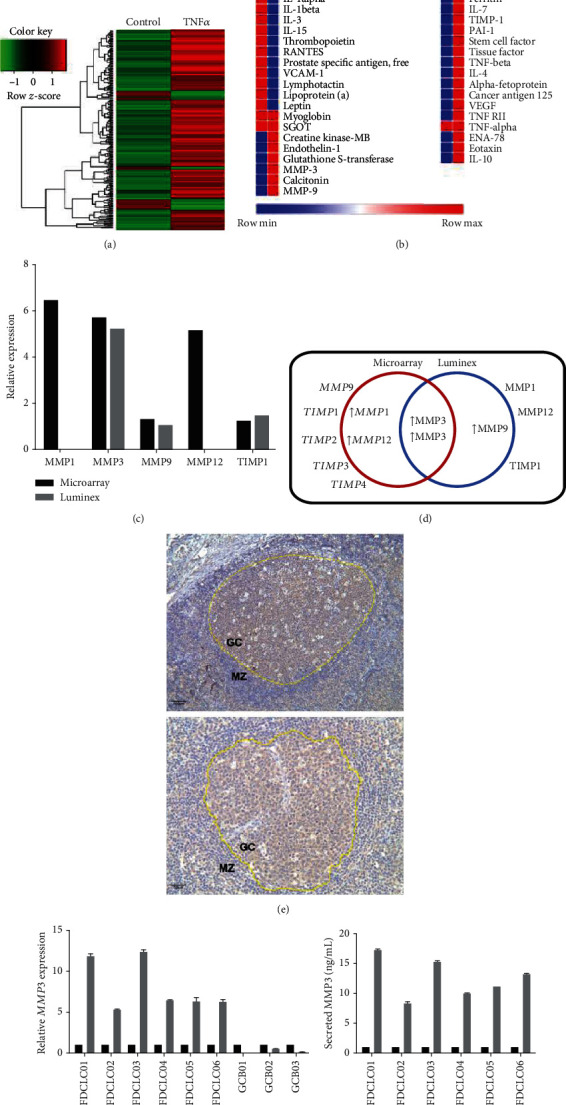
Human follicular dendritic cell-like cells (FDCLCs) in germinal centers (GCs) express MMP3. (a) Microarray gene expression profiling analysis comparing nontreated control and TNF*α*-treated FDCLCs. (b) Luminex protein secretion profiles of the same samples. (c) Microarray and Luminex results for TNF*α*-stimulated FDCLCs. FDCLCs were isolated from resected human tonsil tissues (i.e., from routine tonsillectomies). The isolated cells were treated with 20 ng/mL of TNF*α* for 18 h, and mRNAs and culture supernatants were harvested. Microarray results revealed that TNF*α* exposure increased the expression of *MMP1*, *MMP12*, and *MMP3*, whereas Luminex results indicated that TNF*α* induced the secretion of both MMP3 and MMP9. (d) Schematic depiction of the microarray and Luminex results. Both the mRNA expression and protein secretion of MMP3 were increased. (e) Immunohistochemical staining of MMP3 in human tonsillar sections. Brown color shows MMP3 expression, and blue color indicates stained nucleus (hematoxylin). The boundary between the germinal center (GC) and the marginal zone (MZ) is indicated by a yellow dotted line. (f) *MMP3* mRNA expression in human FDCLCs and GC-B cells. mRNAs were isolated from the TNF*α*-treated cells, and quantitative PCR was then performed. Mmp3 was found to be markedly induced by TNF*α* in all of the examined primary FDCLCs, but not in the GC-B cells. (g) MMP3 secretion of FDCLCs. FDCLCs were treated with 20 ng/mL of TNF*α* for 48 h. MMP3 in the culture supernatant was quantified by ELISA. MMP3 secretion from FDCLCs was markedly induced by exposure of these cells to TNF*α*.

TNF*α* secretion in the GC has been reported previously [21]; therefore, we first investigated the effects of TNF*α* on FDCLCs. Primary FDCLCs were isolated from fresh human tonsils and cultured in the presence of TNF*α*. Both the cells and the culture supernatants were harvested for mRNA expression and protein secretion analyses. We confirmed that TNF*α* induced the expression of various transcripts and proteins in FDCLCs (Figures 1(a) and 1(b)). Among these factors, the expression of MMPs was found to be markedly increased in the presence of TNF*α*. Because the MMPs are indispensable for cell migration and tissue remodeling, we focused on their specific roles in FDCLCs. Subsequent mRNA expression data revealed that *MMP3*, *MMP1*, and *MMP12* transcripts were upregulated in these cells upon exposure to TNF*α*, whereas protein secretion results revealed an increase in the MMP3 and MMP9 levels (Figure 1(c)). Only MMP3 was revealed to be upregulated by TNF*α* at both the mRNA and secreted protein levels (Figure 1(d)). We confirmed that MMP3 is expressed in the human GCs *in vivo* by examining tissue slides of human tonsils (Figure 1(e), the borderline is assigned by the yellow dotted line).

To confirm that MMP3 is produced by FDCLCs and not by GC-B cells, primary FDCLCs and GC-B cells isolated from different donors were analyzed. All of the examined FDCLCs but none of the GC-B cells from different donors showed high MMP3 mRNA expression in the presence of TNF*α* (Figure 1(f)). MMP3 protein secretion from FDCLCs was also increased in the presence of TNF*α* (Figure 1(g)). Taken together, these results indicate that FDCLCs in the GC express MMP3 via TNF*α* signaling.

### 3.2. MMP3 Expression Induced by TNF*α* Is Mediated through ERK1/2 and AP1 Activation in FDCLC

**Figure 2:**
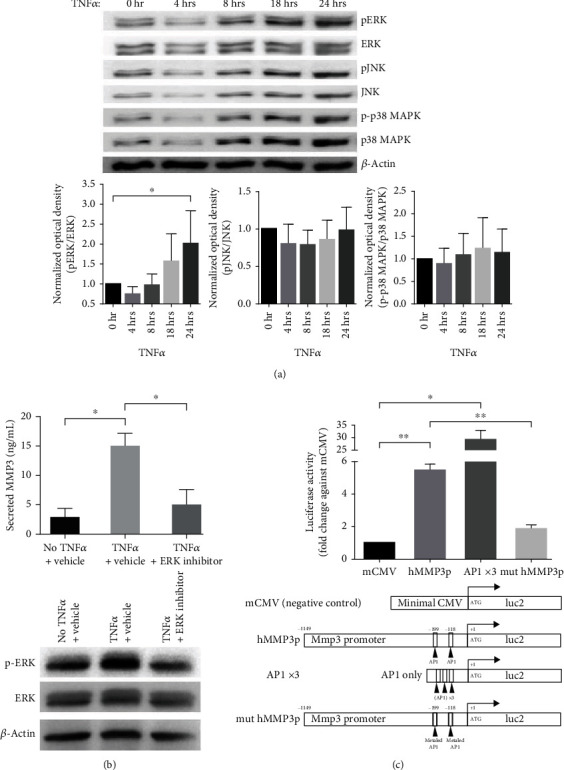
MMP3 expression induced by TNF*α* is mediated through ERK1/2 and AP1 activation in FDCLC. (a) TNF*α* induces ERK1/2 phosphorylation in FDCLCs. Time-course TNF*α*-treated FDCLCs were harvested and lysed, and ERK1/2 phosphorylation was then assessed by western blotting. The ImageJ densitometry plug-in was used for quantitative analysis. Among the MAPKs tested, only ERK1/2 phosphorylation was found to be significantly induced by TNF*α*. (b) TNF*α*-induced MMP3 expression is dependent on ERK1/2 activation. FDCLCs were pretreated with the ERK1/2 inhibitors U0126 and PD98059, and MMP3 secretion in the culture supernatant was analyzed. The inhibition of ERK1/2 phosphorylation was confirmed by western blotting, and MMP3 secretion was analyzed by ELISA. (c) Luciferase reporter assay for the AP1 binding site in the *MMP3* promoter region. Diagrams indicate the vectors used. The MMP3p and AP1 luciferase activity was increased by TNF*α*, but not that of the AP1-mutated Mmp3p construct. Data shown are the mean values with standard deviation (SD) of three independent biological replicates; ^∗^*p* < 0.05.

MMP3 expression has been shown to be regulated at the transcriptional level through the mitogen-activated protein kinase- (MAPK-) AP1 pathway in human chondrocytes and in non-small-cell lung carcinoma cells [22, 23]. To examine if this same signaling pathway also operates in FDCLCs, we assayed the phosphorylation of ERK1/2, JNK, and p38 MAPK following TNF*α* treatment of these cells. TNF*α* significantly increased the phosphorylation of ERK1/2 but had no noticeable effect on the JNK and p38 MAPK phosphorylation status (Figure 2(a)). To confirm that MMP3 expression in FDCLCs is dependent on the ERK1/2 signaling pathway, we examined whether the inhibition of ERK1/2 activation would abolish TNF*α*-mediated MMP3 induction. Treatment of the cells with the ERK1/2 inhibitor U0126 markedly reduced MMP3 expression by TNF*α* to a level comparable with the vehicle-treated (DMSO only) negative control samples (Figure 2(b)). The inhibition of ERK1/2 activation in FDCLCs by U0126 was confirmed by western blotting. These results demonstrated that the MMP3 expression induction by TNF*α* in FDCLCs requires ERK1/2 activation.

The human MMP3 promoter harbors 2 AP1 binding sites [23]. To analyze whether TNF*α*-induced MMP3 production is dependent on these sites, we generated reporter vectors containing the MMP3 proximal promoter with 2 AP1 binding sites (hMMP3p), 3 repeat AP1 sites (cloned from the human MMP3 promoter) without the MMP3 promoter region (AP1 ×3), and an MMP3 promoter-containing mutations in the 2 AP1 sites (mut hMMP3p). A minimal CMV promoter-containing vector (mCMV) was used as the negative control in these analyses. TNF*α* exposure significantly increased the luciferase activity (by 5.3-fold) when using the hMMP3p construct, whereas no noticeable activity was seen with the mut hMMP3p vector (Figure 2(c)). FDCLCs containing the AP1 ×3 construct show even more stronger luciferase signal induction by TNF*α* stimulation. These results indicated that TNF*α*-induced MMP3 promoter activity in FDCLCs is strongly dependent on the activity of AP1 sites in the MMP3 promoter region. Collectively, our data demonstrate that TNF*α* induces MMP3 production in FDCLCs through ERK1/2 and AP1 activation.

### 3.3. FDCLC Migration Is Induced by TNF*α*, which Is Dependent on MMP3 Expression

**Figure 3:**
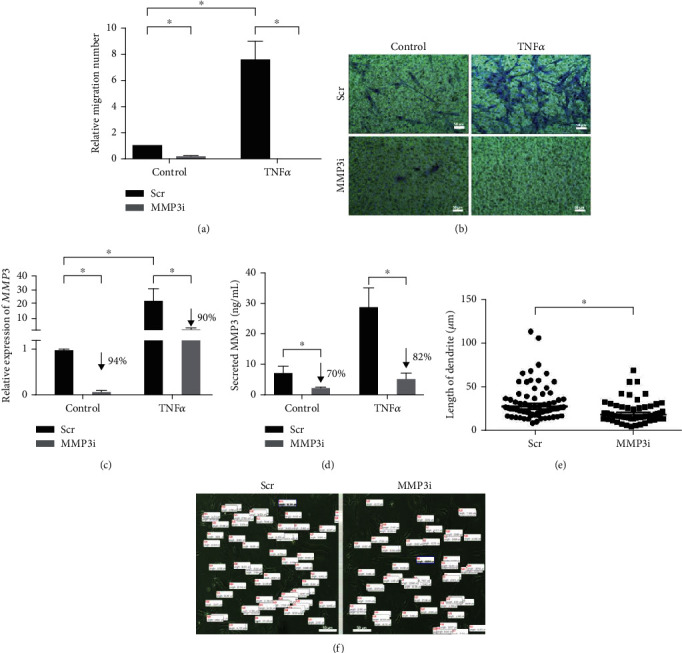
FDCLC migration is induced by TNF*α* and is dependent on MMP3 expression. FDCLCs were transfected with either the MMP3 siRNA (MMP3i) or a scrambled-RNA-negative control (Scr). The cells were then treated with TNF*α*, and a migration assay was performed using A Matrigel-coated Transwell system. (a) Disruption of MMP3 expression drastically suppresses TNF*α*-induced FDCLC migration. The Transwell membranes were stained with hematoxylin and eosin, and the migrated cells that attached to the undersurface of the membrane were counted in 3 different fields under a microscope. Representative images of migrated FDCLCs are shown in (b). MMP3 siRNA significantly reduced *MMP3* mRNA expression (c) and MMP3 secretion (d) in both control and TNF*α*-treated FDCLCs. (e) The length of FDCLC dendrites. MMP3i showed 34% reduction of the length of the dendrites. (f) Representative images of the FDCLC dendrites. Data shown are the mean values with SD of three independent biological replicates; ^∗^*p* < 0.05.

The molecular mechanisms underlying FDCLC migration had previously been uncharacterized. Since MMP3 is known to be involved in tumor cell invasion, we investigated whether TNF*α* induces FDCLC migration and whether MMP3 expression is required for this. We conducted a migration assay with MMP3 siRNA-transfected (MMP3i) or nontargeting scrambled-RNA-transfected (Scr) FDCLCs in the presence or absence of TNF*α* in Matrigel-coated Transwells (Figures 3(a) and 3(b)). TNF*α* stimulation remarkably enhanced FDCLC migration (by 7.5-fold) compared to the controls. The MMP3i-expressing cells, however, showed a robust suppression of TNF*α*-induced migration by 98%. These results strongly indicated that the migration of FDCLCs *in vitro* is induced by TNF*α* but that MMP3 expression is essential for this process.

To verify the effectiveness of our MMP3 knockdown in the migration experiments, MMP3 mRNA expression and protein secretion were measured in Scr- or MMP3i-transfected FDCLCs with or without TNF*α* treatment (Figures 3(c) and 3(d)). TNF*α* exposure increased MMP3 mRNA expression and protein secretion by 22.5-fold and 2.7-fold, respectively, in the Scr cells. The MMP3i cells, however, showed a reduction in these levels by 94% and 70%, respectively. The MMP3i knockdown in the FDCLCs also successfully blocked the upregulation of *MMP3* mRNA expression, and almost completely abrogated the induction of MMP3 secretion, following exposure of the cells to TNF*α*. We additionally found that MMP3i FDCLC had reduced the length of dendrites by 34% on collagen-1-coated plates compared to Scr cells (Figures 3(e) and 3(f)). Overall, these results revealed that TNF*α*-induced FDCLC migration is enhanced by MMP3 expression, and the TNF*α*-mediated MMP3 expression is dependent on ERK1/2 and AP1 activation.

## 4. Discussion

A prolonged exposure to TNF*α* and GC spreading are characteristic features of many lymphoid lesions, including autoimmune diseases and some lymphomas. The molecular mechanisms that control the migration of FDCs, which are unique stromal cells in the GC, have not been actively investigated to date due in part to the lack of an adequate FDC study model. In our current study, however, we have now successfully explored the molecular pathways underlying the TNF*α*-induced migration processes of stromal cells in the B cell follicle by utilizing unique human primary FDCLCs that have been reported to retain some of the physiologic properties of normal FDCs [18, 24].

We found from our present analyses that MMP3 is expressed in the GCs *in vivo* and that FDCLC migration is dependent on this expression *in vitro*. The expression of MMP3 in FDCs is mediated through the ERK1/2-AP1 signaling axis by TNF*α*, which is known to be produced by B cells in the GCs [25]. TNF*α* can activate multiple signaling pathways, including three MAPKs, i.e., ERK1/2, JNK, and p38 MAPK [26, 27]. These kinases mainly induce the transcription factors NF-*κ*B or AP1 [28, 29]. AP1 is closely associated with TNF-mediated inflammatory responses, including chemokine and cytokine production [30]. In our current study, we found that TNF*α* increases the activity of AP1 through ERK1/2 phosphorylation in FDCLCs. AP1 is bound to the *MMP3* promoter region and induces MMP3 expression. These results are consistent with those of previous studies in other various cell types, which showed that ERK1/2 and AP1 control the expression of diverse MMPs. MMP3 expression has been shown to be induced through the TNF*α*-MAPK-AP1 pathway in nucleus pulposus cells and synovial fibroblasts [14, 15]. In addition, MMP9 is increased by TNF*α*-ERK1/2-AP1 in human trophoblastic cells [31]. TNF*α* also induces MMP1 expression in human fibroblasts through MAPK-AP1 signaling [32]. Similarly, the expression of MMP13 is mediated by TNF*α* in human articular chondrocytes and is dependent on ERK1/2 and AP1 [33]. Further investigations are needed, however, to look into the expression changes of other MMPs and TIMPs in FDCLCs to uncover more details of the molecular mechanisms of FDCLC migration.

Another interesting finding from our present experiments was that FDCLCs with an MMP3 knockdown appear to have shorter dendrites than normal cells on collagen-1-coated plates (Figures 3(e) and 3(f)). Similar findings have been described in a previous study which reported that FDCs isolated from the mouse GCs attached to a collagen-1 matrix, spread out, and generated FDC networks *in vitro* [34]. Collagen-1 is the major stromal collagen subtype of human GCs, and MMP3 can degrade collagen-1 through the conversion of MMP1 from its proform to its active form [35–37]. Our current results have revealed that an MMP3 knockdown suppresses TNF*α*-induced FDC migration. Taken together, our present data and previous evidence now indicate that FDC dendrite elongation in the GC and migration likely require the degradation of collagen-1 through a complex interaction of MMPs mediated via TNF*α* signaling from GC-B cells.

In conclusion, this is the first study to our knowledge to reveal the core molecular mechanisms underlying FDCLC migration. Our findings can be a basis for further delineation of FDC-GC biology for specific Ab production, and for the development of targeted treatments for GC-related disorders including follicular lymphoma and autoimmune diseases.

## Data Availability

All data are available on request from the authors (H-KP: ledmilage@gmail.com; C-SP: csikpark@amc.seoul.kr).
